# *Agave atrovirens* fibers as substrate and support for solid-state fermentation for cellulase production by *Trichoderma asperellum*

**DOI:** 10.1007/s13205-016-0426-6

**Published:** 2016-05-21

**Authors:** Naivy Y. Nava-Cruz, Juan C. Contreras-Esquivel, Miguel A. Aguilar-González, Alberto Nuncio, Raúl Rodríguez-Herrera, Cristóbal N. Aguilar

**Affiliations:** 1 Food Research Department, Faculty of Chemistry, Universidad Autónoma de Coahuila, 25280 Saltillo, Coahuila Mexico; 2 CINVESTAV-IPN, Unidad Saltillo, Ramos Arizpe, Coahuila Mexico

**Keywords:** Selection, Pretreatment, Biodegradation, Enzyme activity, Cellulase

## Abstract

Many efforts have been made to produce cellulase with better features and conditions, and filamentous fungi have played an important role in the bioprocess, growing in liquid and solid cultures with sugarcane bagasse, corn stover and others lignocellulosic materials. In the present study, *Agave atrovirens* fibers were partially characterized, thermal pretreated and used as support, substrate and inducer source for cellulolytic complex production by four strains of the genus *Trichoderma*, where *T. asperellum* was selected as the best option for this process after evaluating the enzyme activity and the invasion capacity on the pretreated *Agave* fibers. Fungi were able to grow on the *Agave* fibers secreting the complex cellulolytic enzyme. Results show *Agave* fibers as a good carbon source and support for *T. asperellum* for the production of the cellulolytic complex (endoglucanase 12,860.8 U/g; exoglucanase 3144.4 U/g; and β-glucosidase 384.4 U/g). These results show the promising potential this material could have in the production of the active enzyme cellulase complex.

## Introduction

The *Agave*, a native plant of Mexico, grows in desert and semi-desert areas. In 2012 the sown area in Mexico was about 137,626.27 ha and 19,876.07 Ha only were harvested, which gives a production of 1,686,337.41 t of *Agave*, with a price of 96 dollar/t (SIAP 2012). The main use of this crop is for beverages production, such as Tequila, Mezcal and Pulque, but this is only with some species like *Tequiliana weber* var azul and *Angustifolia* per example. A minority of these plants are used to make knitting yarns, paper and as limiting barrier in some areas; certain towns use the leaves to cook traditional Mexican dishes. As a result a large quantity of these plants have no use in the area. When the *Agave* flower appears it can reach 10 m high and after that the *Agave* plant dies, because this flower consumes all the disposable sugars present. So it is necessary to emphasize the importance in making use of the produced *Agave* crops that nowadays does not have a real use, though it could be used to obtain considerable amounts of fermentable sugars, oligosaccharides, composite and diverse materials, in addition to the alcoholic beverages.

To obtain many of these mentioned products, it is necessary to degrade the complex fibers of the *Agave* leaves.

The Agave cell wall composition is structured mainly by cellulose and hemicelluloses that can be hydrolyzed to simple sugars the action of fermentation. The third major component in the Agave cell wall is lignin, which has a negative effect in the hydrolysis of polysaccharides and is the main cause of the recalcitrance of the cell wall, that is the resistance of cell walls to be converted into fermentable sugars. The percentages of these three main components is variable between crops. For example, the cellulose percentage may vary from 65 to 85 between different species. The hemicellulose content could be from 3 up to 11 % in some species. And the lignin can be present in different species from 7 up to 16 % (Escamilla-Treviño 2012; Nava-Cruz et al. 2015).

Other important components of the cell wall in Agave plants, mainly in the leaves are the water-soluble carbohydrates, also called non-structural carbohydrates, which are released after thermal treatment. In some species, such as *Agave tequiliana weber*, these components can be present in 90 % of the dry matter (Mancilla-Margalli and Lopez 2006). In this kind of carbohydrates, fructans is the main component of the polymers. There is also present in this complex wall, the non-structural sugars, but these in much lower levels decreasing from the base to the top.

One of the most promising ways is the use of cellulases, which have the capacity to hydrolyze such leaves, resulting in fermentable sugars that can be used in different ways. For that reason the production of a cellulase complex using friendly ways that does not compromise all the factors involved is necessary. Cellulose is a polymer of glucose bound by β-1,4 linkages. But not all the cellulose materials have the same chain length and the level of interaction between chains (Cowling 1975). In waste cellulose between 40 and 60 % is cellulose, the rest are divided in hemicelluloses, lignins and other residual materials (Eveleight et al. 2009). The use of lignocellulosic materials for this objective is also increasing. These materials are cheap, abundant and renewable; all these features make the material appropriate to use as substrate in the fermentation process to produce cellulases (Maeda et al. 2011).

Nowadays, there is a crucial need to produce cellulases with better specifications and through natural materials and ways. Cellulases hold the third place worldwide in enzyme industry by dollar volume (Rani et al. 2010). This group of enzymes are formed by three single enzymes: endoglucanases, exoglucanases and β-glucosidases; the first ones hydrolyze the cellulose polymer exposing reductive and no reductive ends of the linear polymer of glucose, while the second ones attack these terminations to liberate cellobiose and cellooligosaccharides (Bansal et al. 2011; Deswal et al. 2011; Talebnia et al. 2010). The β-glucosidases (BGL) will join the cellobiose units to liberate finally the desired product, glucose (Sukuruman et al. 2009; Yah et al. 2010). Degradation process of cellulose consists of a six-step complicated process; the last one is a uniform catalysis process that includes participation of β-glucosidase on cellobiose (Chauve et al. 2010).

The interest in producing cellulases using new sources is increasing and new catalytic features are required. Several microorganisms capable of degrading the cellulosic material have been reported; actually there is a variety of studied microorganisms with special features such as aerobic and anaerobic bacteria (Gilkes et al. 1991; Kumar et al. 2004; Thirumale et al. 2001) soft and white rot fungi (Chung-Yi et al. 2009; Lo et al. 2010; Shrestha et al. 2009; Tanaka et al. 2009) and anaerobic fungi (Dashtban et al. 2009; Ljungdahl 2008). In some cases the cellulases are secreted as free molecules, like the example of filamentous fungi, actinomycetes and aerobic bacteria. The most used microorganisms to produce these kinds of lignocellulosic enzymes are filamentous fungi such as *Trichoderma, Fusarium, Phanerochaete, Penicillium*, etc. (Bak et al. 2009; de Siqueira et al. 2010; Javed et al. 2007; Lo et al. 2010; Mathew et al. 2008). The aim of this study was to produce cellulases in solid-state fermentation using as unique carbon source, inducer and support, *Agave atrovirens* fibers, by the action of a strain of *Trichoderma* spp.

## Materials and methods

### Fungal strains and materials

A set of 4 strains of *Trichoderma* identified as *Trichoderma harzianum* (T1-04), *Trichoderma asperellum* (T2-31) and 2 different more only known as *Trichoderma* spp. codified as *Trichoderma* T2 and *Trichoderma* T2-11 were obtained from the micoteca of the Autonomous Agrarian University Antonio Narro, and the T2 were obtained from the micoteca of the Autonomous University of Coahuila, both in Saltillo Coahuila, Mexico. The strains were reactivated in PDA at 28 °C until they have enough spores to make a suspension with Tween 80.

The *Agave* plant was collected also in the first University. Potato agar dextrose (PDA) was purchased in Sigma Aldrich as well as carboxymethyl cellulose (CMC), *p*-nitrophenyl, β-d-glucopyranoside (pNPG) and the compounds of the Mandels medium. Paper filter strips of 1 × 5 cm (50 mg, Whatman #1), citrate buffer pH 4.8, 0.05 M were also used in this study.

### *Agave* fiber characterization

The fibers of *Agave atrovirens* were characterized as previously reported by (Medina et al. 2011) to measure the cellulose content by a gravimetric technique with the acid and neutral detergent fiber method (Van Soest et al. 1991) with two different types of fibers; the first one is a hydrothermal treatment and the second one a chemical treatment with NaOH. In the hydrothermal treatment the fibers were peeled and sliced into small squares (2 × 2 cm approximately), then the pieces were autoclaved for 30 min to 120 °C. After that, the material was dehydrated in an oven to 70 °C for 48 h. The reducing sugars present in the fibers were partially removed by multiple washing cycles with boiling water. The fibers were dried and then milled in a grinder (Mussatto et al. 2008; Flores-Maltos et al. 2014).

For the chemical treatment the fibers were peeled and sliced into small squares (2 × 2 cm approximately) and dehydrated in an oven at 70 °C for 48 h. The material was then milled in a grinder and placed in an NaOH solution (2 %) in a relation of 1:20. This was autoclaved by 30 min at 120 °C. The material was washed, filtered and dehydrated at 70 °C (Medina et al. 2011).

The cellulose content was evaluated by a gravimetric technique (neutral detergent fiber method) using 0.5 g of the sample heated to boiling in 100 ml of neutral detergent plus 50 µl of heat stable amylase added before the beaker is placed on heat. At this point sodium sulfite is added (0.5 g). Then the sample is boiled for 1 h and filtered on sintered glass coarse crucible or Whatman 54 paper.

In the acid detergent fiber (ADF) the procedure was according to the AOAC. This procedure avoided the use of decalin. The Klason lignin procedure was developed at the same time.

For both kind of fibers were also measured the total and reducing sugars with citrate buffer 50 mM at pH 4.8 and 50 °C (Trevelyan and Harrison 1952; Miller 1959), respectively, and the pH.

### *Agave* fibers pretreatment

In this part were used the last two procedures described above (hydrothermal and chemical) to evaluate which one gives the best results in growing and enzyme activity, so hydrothermal and chemical procedures were evaluated for strains (T2-31, T1-04, T2-11 and T2). Radial growth and enzyme activity were evaluated to choose the best pretreatment of the fibers.

### Strain selection

Kinetics were run for the four strains of *Trichoderma* (previously mentioned) to observe the radial growth and find which strain can adapt more easily to the *Agave atrovirens* fibers. The fermentations were realized in 6-cm-diameter petri dishes to observe the radial growth, using 3 g of dried fibers and adjusted to 80 % humidity with the Mandels medium (NH_4_)_2_ SO_4_ (1.40), KH_2_PO_4_ (2.03), CaCl_2_ (0.30), MgSO_4_·7H_2_O (0.30), peptone (1.0), and a trace metals solution from FeSO_4_·7H_2_O (0.005), MnSO_4_·4H_2_O (0.0016), ZnSO_4_·7H_2_O (0.0014), CoCl·6H_2_O (0.02), urea (0.3), yeast extract (0.25), CuSO_4_·5H_2_O (0.001) (in g/l), and for this stage, the dishes were inoculated with an explant (1 cm diameter) of a previously reactivated strain for each of them. The kinetics were stopped when the first strain reached the limit of the Petri dish (84 h).

With respect to radial speed of growth, the distance reached after 12 h for each strain to the cardinal points (North, South, West, East) were measured and then the velocity per strain was calculated.

At the end of the fermentation (84 h) enzymatic activity for the three components of the complex (exoglucanase, endoglucanase and β-glucosidase) to the obtained extract was measured; protein and total sugars were also measured as is described below. Thereby, the best enzymatic activity titers and the radial growth were used as criteria for choosing the strain to be used in the fermentation.

### Growth analysis

To observe the growth pattern presented by the selected strain of *Trichoderma* in the *Agave* fibers, and the adaptation level to the substrate and support, scanning electronic photomicrographs of the initial and final time (84 h) of the first fermentation for radial growth were taken. The samples were taken directly from the reactor, using 1 g of fibers in a thermo-balance at 110 °C until the samples were dried. The samples were placed in aluminum slides and subjected to an environmental scanning electron microscope (Phillips XL30-ESEM) with low vacuum at 20 keV (kilo electron volts) with a distance of 7.5 mm and a spot size of 4.5.

### Time course of cellulase production in SSF

The strain with best results in the previous stages was selected to realize a kinetic and observe the time with the best enzyme activity. The conditions for the fermentation were the same as used in the selection fermentation, and the fibers were also adjusted to 80 % humidity with Mandels medium and incubated at 29 °C for 312 h. An inoculum of the spore suspension obtained from a previous strain reactivation (1 × 10^7^ per gram of fibers) was added to the fibers. To collect the samples each 24 h 14 plates (reactors) in triplicate were assembled.

### Cellulase production in SSF bioreactors

The enzyme production was realized using 1 kg fermentation trays, and using as support and substrate *Agave atrovirens* fibers (200 g dry weight). The humidity was adjusted at 80 % as before with Mandels medium. This reactors were performed three times corresponding to the highest enzyme activity points. An inoculum of the spore suspension (1 × 10^7^/g of fibers) was added in the medium, obtained from the previous reactivation (7 days), all this material was carefully homogenized and incubated at 29 °C for 216, 240 and 312 h to obtain the best titers in each enzyme.

### Crude extract recovery

For the strain selection 20 ml of citrate buffer (pH 4.8, 0.05 M) was used for each tray and was carefully homogenized for 2 min, and separated with a muslin cloth. Thereby the obtained extract was kept in congelation for further analysis. For obtaining the enzymatic extract, 1 kg of fermented mass from fermenter trays was put under manual pressure without any additional buffer. The fermented material was put in a muslin cloth and squeezed, the obtained liquid stored in congelation for further analysis.

## Analytical determinations

### Exoglucanase activity

Exoglucanase activity was analyzed according to the Roussos modification method (1985); Whatman filter No. 1 paper strips (substrate) were collocated in tubes with 1 ml of the buffer and 1 ml of the sample (reaction mix), it was incubated at 50 °C for 1 h in a thermo bath, and then reducing sugars were determined with Miller’s method (1959). An enzyme blank and a substrate blank were used, the first one contained the substrate (1 strip) and the buffer (1 ml), and the second one contained the enzymatic extract (1 ml) and the buffer (1 ml). To calculate the final absorbance, the blanks were added themselves and then subtracted from the reaction mix; after that formula (1) was used to calculate the international units of enzymatic activity. One enzyme activity unit was defined as the amount of enzyme that released 1 µmol of glucose per minute under the assay conditions.

1 $$\frac{U}{L} = \frac{g}{L} \times \frac{\text{vol rxn}}{\text{vol ext enz}} \times \frac{{1 {\text{mol}}}}{{180\frac{\text{g}}{\text{mol}}}} \times \frac{{1 \times 10^{6 } \mu {\text{mol}}}}{{1 {\text{mol}}}} \times \frac{1}{\text{rxn time}}$$

vol = volume; rxn = reaction; ext = extract; enz = enzyme.

### Endoglucanase activity

This activity was performed using Roussos adaptation method (1985). It consists in a reaction mix of 1 ml of CMC (1 % in citrate buffer pH 4.8, 0.05 M) and 1 ml of the enzymatic extract. It was incubated in a thermo bath for 30 min at 50 °C, and after that reducing sugars were determined to the samples with Millers method. The international units of enzymatic activity were calculated by the same Eq. (1). This assay also used the enzyme and substrate blanks.

A qualitative analysis was also performed to prove endoglucanase activity, using CMC (0.2 %) in a citrate buffer (0.05 M, pH 5) with agarose (1 %) in petri dishes. The extract (50 μl) was placed in the middle of the solidified mixture and the reaction was maintained for 10 min. After that, the plate was incubated at 37 °C for 12 h. The plate was washed with distilled water ten times, with 10-min rest between washings. The dish was covered with Congo red (0.1 %) and left for 1 h, after that it was washed with NaCl (1 M) five times, giving 10 min between washing.

### β-Glucosidase activity

This activity was determined by Vattem and Shetty method (2002). 100 µl of p-NPG (9 Mm) was used as substrate, then 100 µl of the crude extract with 800 µl of the citrate buffer (0.05 M, pH 4.8) was added. Mix reaction, substrate and enzyme blanks were also used as previously. All assays were performed in triplicate.

### Quantitative enzyme assay

A 0.2 % carboxymethylcellulose (CMC) in citrate buffer 50 mM with pH 5 was prepared. Agarose (1 %) was added and placed in petri dishes to form a gel. 50 µl extract fermentation were located in the middle of the plate and after 10 min were incubated at 37 °C for 12 h in humidity presence to avoid dehydration. The sample was washed with distilled water at intervals of 10 min between each washing. The sample was dyed with Congo red (0.1 %) and after 1 h the sample was washed with NaCl 1 M to remove excess.

### Other analysis

The extracts obtained from the fermentation were also analyzed for protein using the Bradford method (1976), and for total sugars using the Dimler method (Dimler 1952). All assays were performed in triplicate.

## Results

### *Agave* fiber characterization

Table 1 summarizes the results of the different analysis realized to the pretreated fibers. The content of cellulose found in the *Agave atrovirens* fibers was about 23.48 % with hydrothermal treatment, and 67.12 % w/w for the chemical treatment. The total sugars found in the hydrothermal one were 0.16 and 0.73 g/l in the chemical one. The reducing sugars are 0.04 and 0.07 g/l for hydrothermal and chemical, respectively. The pH was 5.40 for the hydrothermal and 6.56 in the chemical one.

**Table 1:** Partial characterization of the Agave atrovirens fibers

Treatment	Cellulose (%)	Total sugars (g/l)	Reducing sugars (g/l)	PH
Hydrothermal	23.48	0.16	0.04	5.40
Chemical	67.12	0.73	0.07	6.56

### *Agave* fibers pretreatment

The chemical pretreatment did not have influence on the microbial growth in the fibers with any strain and in consequence the enzyme activity was not expressed. The hydrothermal pretreatment shows growth with the four strains, so for this reason, the selected pretreatment was the hydrothermal one. This pretreatment results in a bigger surface area for the enzymatic system induced for the carbon source through *Trichoderma.*

### Strain selection

The radial growth for each strain was measured and the average in the growth rate obtained. Enzymatic activity was also tested. The results are given in Table 2.

**Table 2:** Cellulase production in SSF bioreactors

Enzyme	Time (h)	Enzymatic activity (U/g)
Exoglucanase	240	3144.4 ± 87.4
Endoglucanase	216	12,860.8 ± 186.3
β-Glucosidase	312	384.4 ± 26.6

The growth rate did not represent a significant difference between strains, the four strains were able to grow in the fibers with similar speed, causing the excretion of cellulolytic enzymes by the strains.

The enzymatic activity presented by *Trichoderma asperellum* (T2-31) at 84 h was higher than the other strains, with 606.4 U/g of exoglucanase, 1213.2 U/g of endoglucanase and 582 U/g of β-glucosidase at 84 h. These results are very high compared with the other three strains, which make a suitable strain to the next steps in the investigation. This shows its capacity to degrade the fibers in a better way for the secretion of enzymes with good cellulolytic activity.

### Growth analysis

**Fig. 1:**
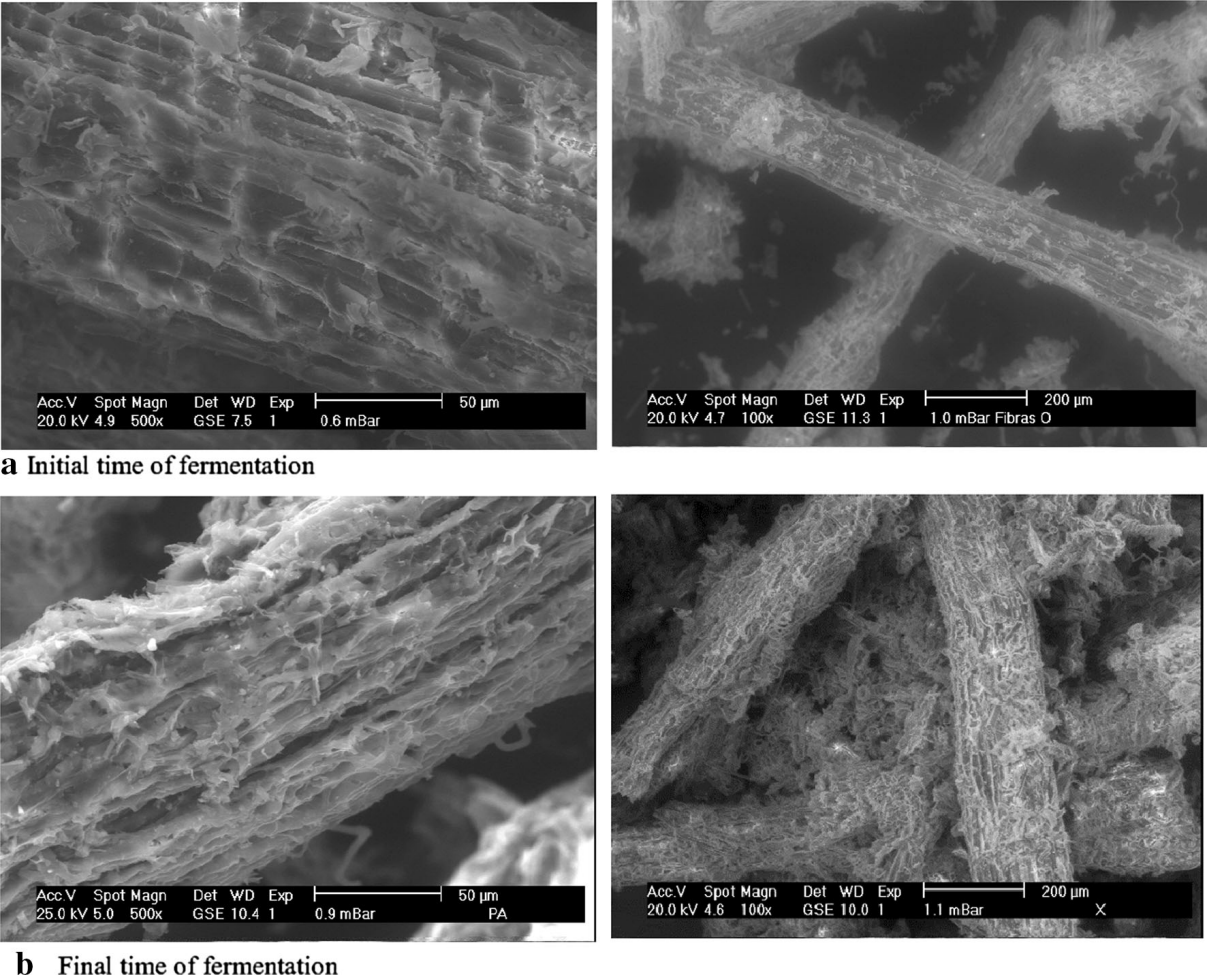
Growth analysis of *Trichoderma asperellum* on *Agave atrovirens* fibers with a hydrothermal pretreatment

In Fig. 1a can be seen the *Agave* fibers without growth, while in Fig. 1b the *Agave* fibers appear wrapped by mycelium after 84 h of incubation under the conditions mentioned before. Only the images of *Trichoderma asperellum* T2-31 are shown in the agave fibers, since it was the strain with better features in growing and enzyme activity in comparison with the other three.

These micrographs demonstrate the capacity of *Trichoderma* to grow using the *Agave* fibers as substrate and support.

### Time course and cellulase production

**Fig. 2:**
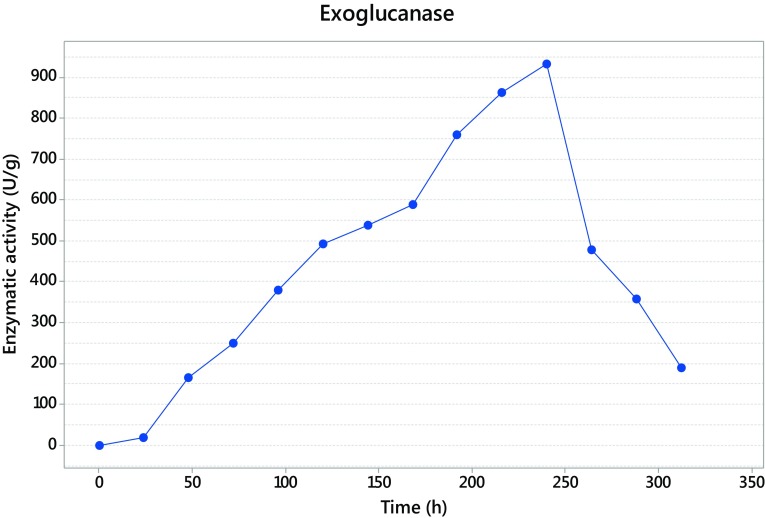
Exoglucanase activity

**Fig. 3:**
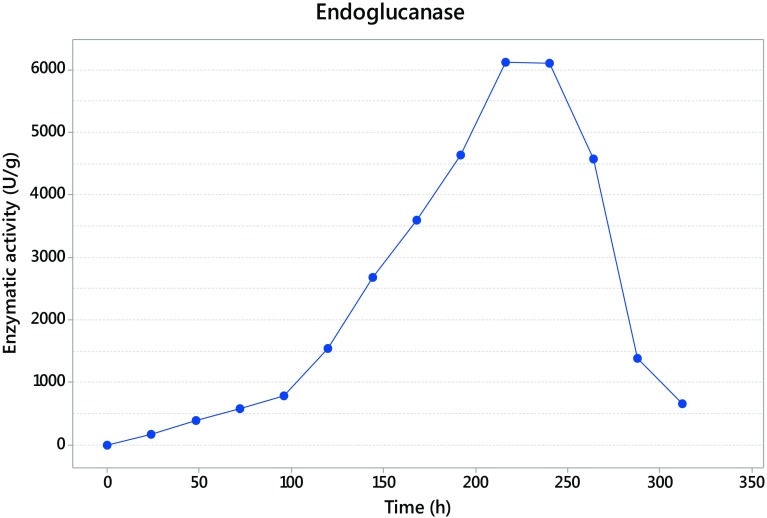
Endoglucanase activity

**Fig. 4:**
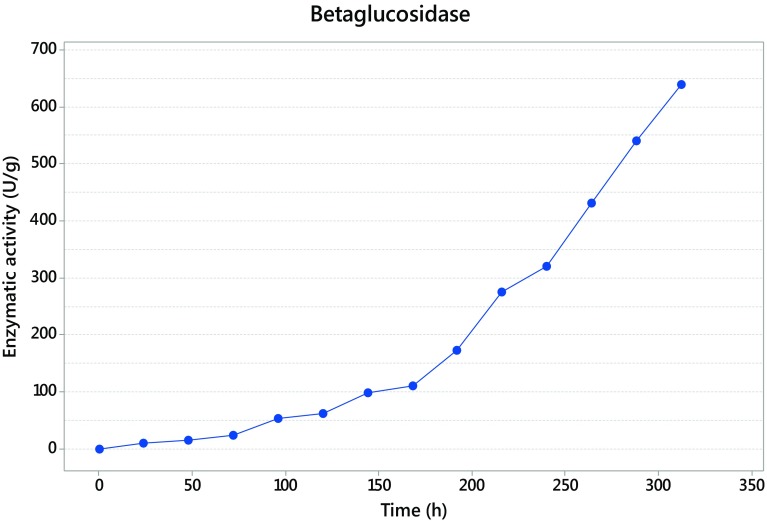
β-Glucosidase activity

The fermentation with *Trichoderma asperellum* was maintained until 312 h, and the sampled each 24 h. The maximum activity was detected at 240 h for exoglucanase with 932 U/g (Fig. 2), while endoglucanase presented its maximum activity at 216 h giving as result 6116 U/g (Fig. 3), and finally the β-glucosidase presented at 312 h with 640 U/g (Fig. 4).

**Fig. 5:**
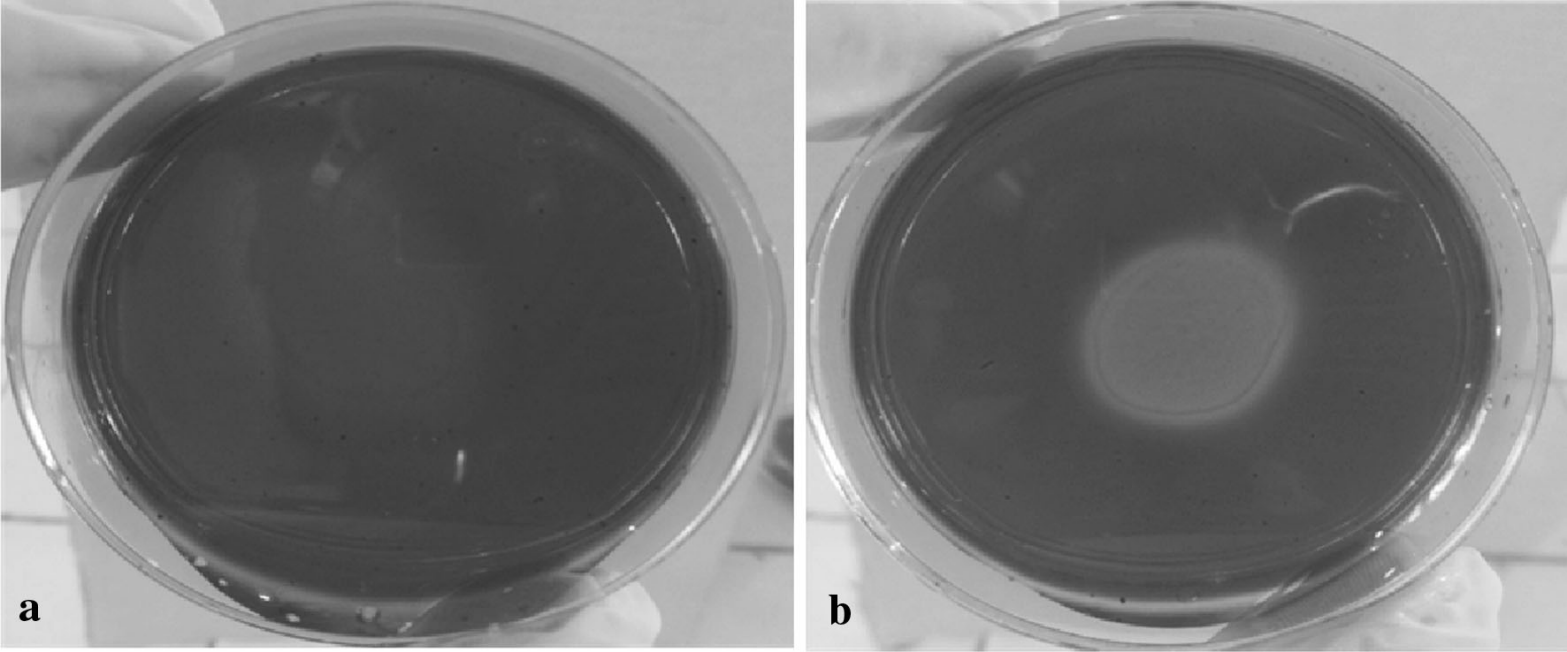
Qualitative enzyme activity. Carboxymethylcellulose plate dyed with Congo red. **a** Blank (without fermentation extract). **b** Sample with fermentation extract in the surface

In Fig. 5 can be seen (part a) the blank of the assay that is completely red, and then the hydrolysis made by the enzymatic complex of *Trichoderma asperellum* (part b), where it is clear the hydrolysis halo marked by the discoloration of the Congo red dye.

### Cellulase production in SSF bioreactors

Since the times of highest enzyme activity are known, the solid-state fermentation was performed in bigger reactors, using aluminum trays of 1 kg. One tray for each of the enzymes was collocated and stopped at different times (216, 240 and 312 h). The tray of 216 h gave 12,860.8 U/g of endoglucanase, the fermentation stopped at 240 h showed 3144.4 U/g of exoglucanase and the fermentation at 312 h give 384.4 U/g of β-glucosidase (Table 2).

### Quantitative enzyme assay

In Fig. 5 it can be seen in part (a) the blank of the sample were no extract cocktail fermentation was added, and all the carboxymethylcellulose plate are intact. In part (b) it can be seen how the extract fermentation cocktail acts in the carboxymethylcellulose degrading the surface, evidenced by the Congo red dye.

### Other analysis

**Fig. 6:**
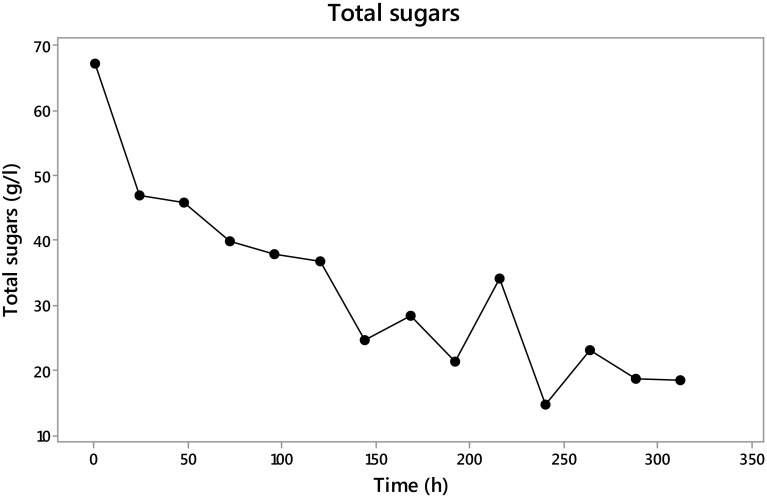
Total sugars during fermentation with *Trichoderma asperellum*

The total sugars quantified by the Dimler method were present in 67 g/l at the beginning of the fermentation and at 312 h were only present with 18 g/l (Fig. 6). These data show the sugar intake by *Trichoderma*, using up an average of 20 g/l in the first 24 h and after this 4 g/l per day. A slight increase in the total sugars present in the extract could be seen at 216 h with 9 g/l more than the previous sampling followed by the consumption of 20 g/l in the next 24 h, and then another increase of 8 g/l with a normal previous tendency of 4 g/l each 24 h.

**Fig. 7:**
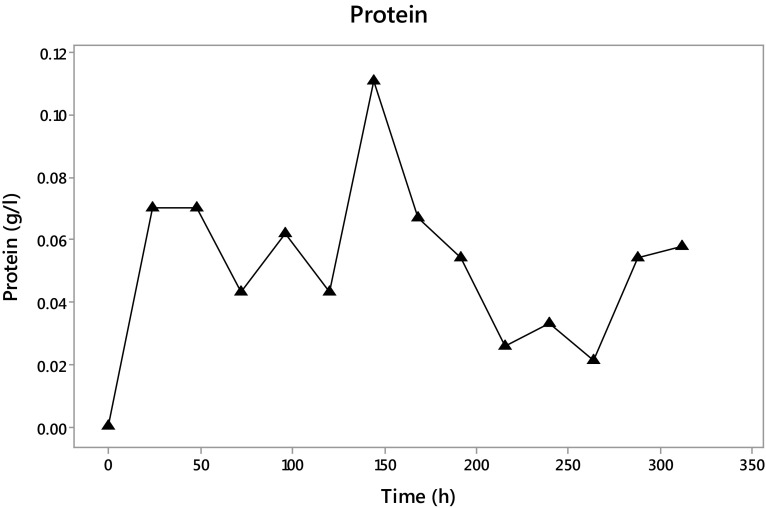
Protein content in the fermentation with *Trichoderma asperellum*

The protein quantification (Fig. 7) shows that at 144 h of fermentation the maximum protein in the extract is present with 0.111 g/l, followed by a decrease until 0.058 g/l at the end of the fermentation.

## Discussion

The percentage of cellulose found is higher in the NaOH-pretreated fibers and this should be for the removal of interfering compounds with the alkaline treatment, which expose widely the inside content (Medina et al. 2011). Martin et al. (2006) reported 57.9 % w/w cellulose in *Agave fourcroydes* which is similar to that reported here, while Caspeta et al. (2014) reported 43 % cellulose in *Agave* bagasse. The amount found is influenced by the used method.

In the characterization of the *Agave* fibers, the total and reducing sugars in the chemical treated fibers are higher than the hydrothermal ones, owing to the opening in the fibers structure that allows the release of more compounds, including carbohydrates as pentose (Hartree et al. 1987; Kumar et al. 2008).

The pretreatment selected to the fibers was the hydrothermal one due to the zero growth with the chemical one, probably caused by the formation and release of interferential compounds with the NaOH interaction with the fibers, as saponins, flavonoids ant others Güclu-Üstündag and Mazza (2007).

The hydrothermal pretreatment given to the fibers changes the crystalline unreactive form of the cellulose to an amorphous structure that facilitates the access to the cellulose material. This benefits the accessibility to the enzyme complex because of the disruption of the cellulose–hemicellulose–lignin structure and stirring compounds (Kumar et al. 2008).

In the strain selection analysis presented in Table 3, it can be seen the strain T2-31 with very high levels of endoglucanase compared with the other three strains, the same result but not with such difference with the exoglucanase and β-glucosidase is shown. This could be probably explained because this specie presents a better adaptability to the agave fibers in order to a synergic enzyme release of the three components of the cellulolytic complex, which makes in set the best performance of this four strains of *Thrichoderma.* The suitable features of *Trichoderma asperellum* have been also reported in many biochemical processes because of its enhanced cellulose production and lower catabolite repression. Same features are observed in the present investigation (Raghuwanshi et al. 2014).

**Table 3:** Growth velocity and enzyme activity for the strain selection

Strains	Growth rate (cm/h)	Exoglucanse (U/g)	Endoglucanase (U/g)	β-Glucosidase (U/g)
T2-31	0.038^*^	606.4	1213.2	582
T1-04	0.04^*^	582.8	379.2	537.6
T2-11	0.038^*^	340.4	254.4	157.2
T2	0.032^*^	192.8	58	9.2

* Data statistically similar

Units of β-d-glucopyranose bound by β-(1 → 4) glycosidic links result in the polysaccharide known as cellulose (Kumar et al. 2008). To hydrolyze this polymer, in the fermentation process, the action of three enzymes is necessary that in this study, *Trichoderma asperellum* has shown can produce. This complex is formed by exoglucanase, endoglucanase and β-glucosidase. The time where the three enzymes present the maximum activity corresponds with the common mechanism of the complex enzyme, where the first enzyme appearing is endoglucanase, producing nicks in the cellulose polymer and then the exoglucanase acts to release cellooligosaccharides and finally the β-glucosidase liberate the final product, glucose (Rani et al. 2010). Kellermann and Rentmesiter (2013) and Kumar et al. (2008) reported that in the hydrolysis of amorphous cellulose a ratio of 6:2:1 is commonly seen between endoglucanases, exoglucanases and β-glucosidases, respectively.

The large period of hydrolysis and the prolonged time in the presence of the enzymes could be associated to various factors, one of them, could be a strong binding in the insoluble cellulose (Lan et al. 2013; Zhou et al. 2004). Other option associated to this phenomenon could be an important quantity of enzyme linked in that insoluble cellulose, Zhou et al. (2004) mentioned that, those cellulase adsorbed is similar to the present in the crude extract, and also mentioned that cellulose can be calculated by the difference between the initial protein concentration and the final one. In this study, that cellulose adsorbed was calculated (data not shown) and demonstrate that the cellulose adsorbed, in each one of the three enzymes, was the same as the cellulose analytical calculated. It is important to mark that this work is not optimized and diverse physical and chemical factors such as pH, temperature, adsorption, nitrogen, phosphorous, phenolic compounds and some inhibitors can improve or affect the bioconversion of the lignocellulosic material (Kumar et al. 2008).

Table 4 shows different species of fungi used with the same aim. Raghuwanshi et al. (2014) reported the use of a mutant strain of *Trichoderma asperellum* SR7 under optimized conditions in the degradation of wheat bran with titers of exoglucanase of 2.2, 13.2 IU/g of endoglucanase and 9.2 IU/g of β-glucosidase in periods ranging from 4 to 7 days. These results show the same specie strain but mutated, with titers well below the obtained in the present investigation, revealing the huge capacity of this strain *Trichoderma asperellum* to degrade a very difficult substrate as the *Agave* fibers. The big difference in the period time is important, but still that point, our results shows more than 1000 times more activity in the double of time. There are several species of fungi used for the same purpose, but each one with different production times, therefore a direct comparison is difficult.

**Table 4:** Comparison of cellulase production by *Trichoderma asperellum* with other fungi under SSF

Strain	Substrate	Enzyme activity (UI/g) Exoglucanase	Endoglucanase	β-Glucosidase	References
*Trichoderma asperellum* SR7	Wheat bran	2.2	13.1	9.2	Raghuwanshi et al. (2014)
*Trichoderma harzianum* PPDDN10 NFCCI-2925	Wheat bran	0.74	4.10	–	Pathak et al. (2014)
*Trichoderma reesei* RUT-C30	Horticultural waste	15.0	90.5	61.6	Xin and Geng (2010)
*Aspergillus japonicus* URM5620	Castor bean meal	953.4	191.6	88.3	Nunes et al. (2011)
*Aspergillus niger* FGSCA733	Jatropha curcas seed-cake	3974	–	–	Ncube et al. (2012)
*Fomitopsis sp*. RCK2010	Wheat bran	3.5	71.7	53.7	Deswal et al. (2011)
***Trichoderma asperellum***	**Agave atrovirens fibers**	**3144.4**	**12,860.8**	**384.4**	**Present work**

The challenge in this cellulase production process is the maintenance of optimal conditions to the maximal enzyme production, which needs a balance between diverse factors to avoid the change from a productive state to a nonproductive, that in this case was not yet studied; however, the data shows the capacity of *Trichoderma asperellum* to degrade a complex matrix of cellulose, as *Agave atrovirens* fibers.

The analysis of total sugars suggest that the sugars produced by the saccharification of the *Agave* fibers, was constantly consumed by *Trichoderma asperellum*, which coincide with the time in the enzymes shown its maximum activity, allowing us bearing out that more than the sugars present in the extract, we can find some corresponding to the produced by the enzyme complex.

The tendency in the protein concentration is similar to that showed by the enzyme concentration, suggesting that the enzyme present in the extract was active.

*Agave* fibers of different species have been used to evaluate the effect of various factors present in the saccharification of *Agave* leaves using commercial cellulases or for the production of bioethanol (Li et al. 2014; Medina et al. 2011; Saucedo-Luna et al. 2011). Kalia and Vashistha (2012) reported the use of *Brevibacillus parabrevis* in the modification of the surface of *Agave sisilana* fibers for composite fabrication, and as a result bacterial cellulase treatment improves the features of the material such as thermal and crystallinity, as well as giving a smooth and shiny surface. But they do not report enzymatic titers, since the main objective was the scanning of the *Agave* fiber surface.

## Conclusions

*Trichoderma asperellum* is able to grow using as carbon source and support the *Agave atrovirens* fibers pretreated with a hydrothermal process, releasing an enzymatic complex of cellulase made up by exoglucanase, endoglucanase and β-glucosidase, being the second most active in the degradation of the *Agave* fibers, resulting in promising fermentable sugars.
